# Reward from Punishment Does Not Emerge at All Costs

**DOI:** 10.1371/journal.pcbi.1002868

**Published:** 2013-01-17

**Authors:** Jeromos Vukov, Flávio L. Pinheiro, Francisco C. Santos, Jorge M. Pacheco

**Affiliations:** 1 ATP-group, Centro de Matemática e Aplicações Fundamentais, Instituto para a Investigação Interdisciplinar da Universidade de Lisboa, Lisboa, Portugal; 2 Centro de Física da Universidade do Minho, Braga, Portugal; 3 Departamento de Engenharia Informática & INESC-ID, Instituto Superior Técnico, Universidade Técnica de Lisboa, IST-Tagusparque, Porto Salvo, Portugal; 4 Departamento de Matemática e Aplicações, Universidade do Minho, Braga, Portugal; Princeton University, United States of America

## Abstract

The conundrum of cooperation has received increasing attention during the last decade. In this quest, the role of altruistic punishment has been identified as a mechanism promoting cooperation. Here we investigate the role of altruistic punishment on the emergence and maintenance of cooperation in structured populations exhibiting connectivity patterns recently identified as key elements of social networks. We do so in the framework of *Evolutionary Game Theory*, employing the *Prisoner's Dilemma* and the *Stag-Hunt* metaphors to model the conflict between individual and collective interests regarding cooperation. We find that the impact of altruistic punishment strongly depends on the ratio *q/p* between the cost of punishing a defecting partner (*q*) and the actual punishment incurred by the partner (*p*). We show that whenever *q/p*<1, altruistic punishment turns out to be detrimental for cooperation for a wide range of payoff parameters, when compared to the scenario without punishment. The results imply that while locally, the introduction of peer punishment may seem to reduce the chances of free-riding, realistic population structure may drive the population towards the opposite scenario. Hence, structured populations effectively reduce the expected beneficial contribution of punishment to the emergence of cooperation which, if not carefully dosed, may in fact hinder the chances of widespread cooperation.

## Author Summary

Altruistic punishment — when a cooperative individual pays a cost to punish her defective partner — has been described as one of the mechanisms that help to explain cooperation's ubiquity in nature. Here, we investigate a model population where individuals interact with each other along the links of a network. The network is built so that it contains the relevant features of real social and biological interaction webs. Individuals engage in cooperation dilemmas with each other and have the possibility to punish defective partners in order to enforce higher cooperation levels. However, it turns out that the introduction of altruistic punishment not always promotes cooperation – in fact, it can actually hinder the spread of cooperation in a variety of cases that we are able to characterize. Effects acting at “micro”, individual level, such as softening the dilemma and reducing the pressure originating from the fear from being cheated and/or the temptation to cheat, can result in lower overall cooperation at a “macro”, population-wide level, due to the complex interference of the social dilemma and the heterogeneous interaction network.

## Introduction

Cooperation, understood as an action which incurs a cost *c* to the individual that performs it, inducing a benefit *b*>*c* to the recipient of that action, is ubiquitous at all levels of biological complexity (i.e. from bacteria to primates) [1]–[3]. However, cooperation requires the existence of an additional mechanism which, at par with it, leads to its evolutionary viability. Up to now, the different mechanisms which were found to pave the way for the emergence of cooperation are inherently “*additive*”, in the sense that two mechanisms, when acting together, enhance the viability of cooperation to emerge, compared to the effect accruing to each mechanism alone [4], [5]. In all cases, what is at stake is the paradoxical collision between individual and population goals. *Evolutionary Game Theory* (**EGT**) [6]–[8] provides an excellent mathematical framework to deal with this challenge and study the evolution of different behaviors in populations.

Two popular metaphors to investigate the emergence and maintenance of cooperation under this framework are the Prisoner's Dilemma (**PD**, widely employed in biology, and applied to many non-human species) and the Stag-Hunt Dilemma (**SH**, very popular in connection with the social contract and other human affairs) [9]–[16]. In particular, the **PD** constitutes the *de facto* prototype metaphor for studies of cooperation. From a game theoretical point of view a rational individual in a two-person *one-shot* **PD** engagement is always better off by not cooperating (defecting), while in real life one often observes the opposite, to a significant extent.

Popular mechanisms that aim at solving this evolutionary conundrum such as kin selection [17], direct reciprocity [13], [18], voluntary participation [19], [20], reputation [21]–[24], social structure [25]–[29], peer and pool punishment [30]–[40], *etc*, are able to promote cooperation by transforming a **PD** into a **SH** [4], [16], [41], [42]. From a sociological perspective, the **SH** portrays a milder dilemma when compared to the **PD**, since it strips *temptation* from the latter, leaving only *fear* in the way between individual and collective interest [43], [44]. Recently, altruistic punishment (which occurs when one individual accepts to pay a cost to impose a higher loss to a peer) was proposed as an efficient mechanism promoting cooperation, based on laboratory experiments showing also that individuals embedded in different contexts punish quantitatively in different ways [34], [45].

Whenever Humans are at stake, one often observes that several mechanisms found to promote, each on its own, the emergence of cooperation, are active simultaneously. Indeed, kin often favor each-other, even in situations in which encounters are repeated, reputation is important and individuals interact and change their minds embedded in population structures well-described by complex social networks. In this context, punishment is no exception.

It is thus important to investigate the impact of altruistic punishment in population environments which are structurally more realistic [46]. Here we explore the evolutionary consequences of altruistic punishment in heterogeneously structured populations for a wide range of the **PD** and **SH** game parameters (see Model section).

We adopt the *scale-free* paradigm [47]–[49] to describe population structure, as it incorporates features which have been found recurrently in many network structures: heterogeneity, in the sense that different individuals, here associated with different nodes of the complex network, may have different number of neighbors, defined by the bi-directional links emerging from them; moreover, the degree distribution, that is, the probability that a given individual has *k* neighbors, follows a power-law dependence 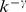. These structures generate, in the population, an asymmetric distribution of wealth and influence [50]–[52] both of which greatly enhance the evolutionary chances for cooperation [28], [46], [53]–[56]. Indeed, in such structures, a few individuals (the hubs) are able to interact with a larger number individuals than the vast majority of the population, somewhat embedded in the spirit of the Pareto principle [57].

In the following we shall study the evolutionary dynamics of structured populations, assessing the role of altruistic punishment in comparison with the corresponding results of the model in which punishment is absent.

## Model

Individuals engage in one-shot games with their first neighbors along the links of a scale-free network (see below) and acquire a fitness associated with the payoff accumulated from all their interactions. Each individual plays unconditionally either as cooperator or a defector. Hence, depending on the strategy pairs, there are four possible outcomes: mutual cooperation yields the reward (*R*), whereas mutual defection results in the punishment (*P*) for both individuals. A cooperative player facing a defector gets the sucker's payoff (*S<P*) whereas the defector earns the temptation (*T*). Following usual practice [25], [28], [44], we set *R* = 1 and *P* = 0 reducing the number of free game payoff parameters to two. Hence, whenever *T>R* = 1 we obtain a **PD** (*T>R>P>S*), whereas *T*<1 gives rise to a **SH** (*R>T>P>S*). Hence under the **PD** rational players are driven into defection both by the temptation to cheat (*T>R*) and by the fear from being cheated (*P>S*), despite the fact that mutual cooperation (*R* = 1) offers a better collective outcome compared to mutual defection (*P* = 0) [11], [43]. Under the **SH**, the tendency to defect derives solely from the fear of being cheated [16], [43], [58].

In our model setup, cooperators have the option to punish defectors by means of peer punishment, that is, a ‘punisher’ pays the cost *q* to induce the punishment *p* on the opposing defector. To keep the analysis simple, we only consider two strategies, punishing cooperators (*C*) and defectors (*D*). In this case, the payoff matrix takes the following form:
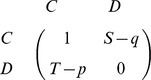
During the evolutionary process, players can adopt the strategy of their neighbors with a probability depending on the payoff difference. In each elementary step, a player *x* is chosen randomly from the population; a second individual *y* is selected at random from the neighborhood of *x*; player *x* adopts player *y*'s strategy according to the pairwise comparison rule [59]–[61], which ascribes the probability 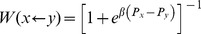 to this process, where *P_x_* and *P_y_* are the accumulated payoffs of player *x* and *y*, and *β* represents the intensity of selection (or alternatively, it measures the errors in decision making and the uncertainty of the strategy adoption process): For high *β* (strong selection) strategies with higher payoff are most likely imitated, whereas for lower *β* values (weak selection), the influence of payoff decreases. No mutations are considered.

Scale-free networks are built according to the *Barabási-Albert* model of growth and preferential attachment. We generated 10^2^ scale-free networks [47] with 10^3^ nodes each and average degree of 4. We computed the average final fraction of cooperators (*x_ffc_*) by averaging the final fraction of cooperators (1 or 0 as the evolution already reached fixation) over a total of 2.5×10^4^ simulations, each starting from an equal fraction of *C*s and *D*s randomly distributed in the network. We took the value *β* = 0.25 for the intensity of selection, a value that optimizes the cooperation levels in scale-free networks in the absence of punishment [62]. This value does not correspond to the *weak selection* limit which we discuss in the following section.

## Results/Discussion

**Figure 1:**
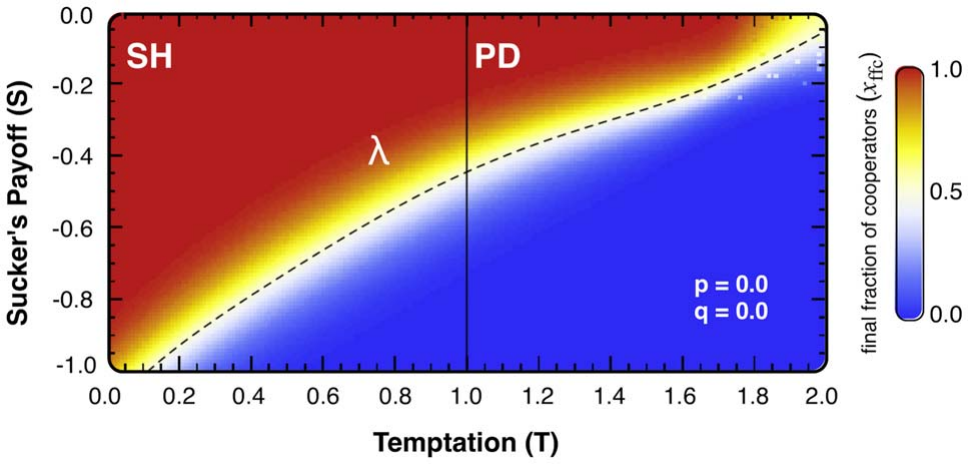
Cooperation level on the *T-S* plane without the punishment mechanism (*p = q = 0*). The dashed line indicates the *edge-curve λ* where the cooperation level reaches 50% (being larger than 50% above the line). Under this scenario that only accounts for the impact of structure in the evolution of cooperation, we obtain *Ω* = 0.49.

We first examine what happens in the absence of punishment (*p = q* = 0), which leaves the network structure as the only mechanism promoting the emergence of cooperation. Figure 1 shows the average final fraction of cooperators on the *T-S* plane in the region associated with the **SH** and **PD** domains (0<*T*<2, −1<*S*<0). We quantify the overall impact of each mechanism in the evolutionary dynamics of cooperation by defining an area-wide *cooperation-index Ω* as the fraction of the area of the *T-S* plane in which *x_ffc_*>0.5. As the decline of the distribution function describing the level of cooperation (displayed in Figure 1) is sharp and the function peaks at 1, the index gives a good measure for the scale of cooperation on average for the payoff parameter region under study. With this definition we obtain *Ω* = 1.0 (*Ω* = 0.0) for overall cooperator (defector) dominance on the whole *T-S* plane, while for the classical result of evolutionary game theory in well-mixed populations we obtain *Ω* = 0.25 (corresponding to half of the **SH** area in the *T-S* plane).

Figure 1 shows the evolutionary outcome on heterogeneous scale-free networks which lead to *Ω* = 0.49, a significant increase of overall cooperation, corroborating previous works [44], [46]. The dashed line shows the threshold where cooperation crosses the 50% mark. In this setting the evolutionary dynamics is mainly hub-driven, given the feasibility of hubs to accumulate a very large fitness. In particular, defector-hubs, which may initially accumulate a high fitness, see their own income decrease in time as they become frequently imitated by their neighbors, leading to a rapid increase of mutual defections in their neighborhood. This dynamics is very different from the reinforcing dynamics induced by a successful cooperator located in a hub, who converts the neighbors to cooperators thereby forming a supporting cooperative cluster [28], [63].

**Figure 2:**
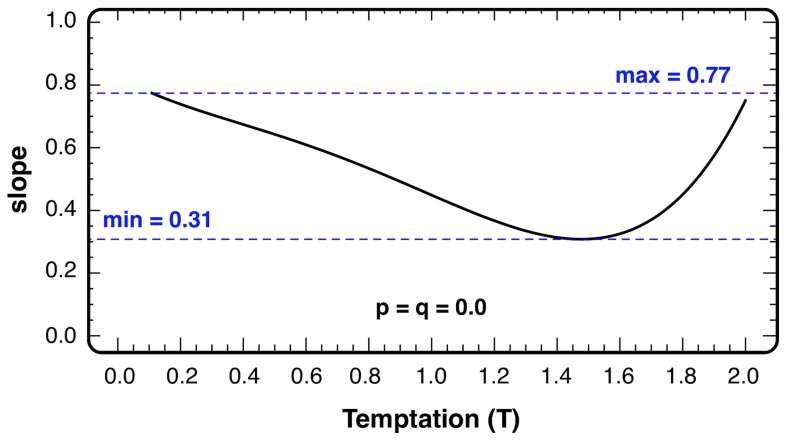
Slope *σ* of the tangent to the edge-curve *λ* (see Figure 1) as a function of the temptation *T* along the 50% cooperation curve (solid line, see main text for details). Blue dashed lines show the upper and lower bounds of the slope function.

The introduction of altruistic punishment induces a shift in the non-diagonal entries of the payoff matrix. This means that the outcome of evolutionary scenarios with punishment can be mapped onto scenarios without punishment for different values of ***T*** and ***S***. Given that the entries are transformed as: ***T→T – p*** **,** ***S→S – q***, punishment amounts to introduce a translation in the *T*-*S* plane defined by the vector with coordinates (*p, q*). The analysis of the slope *σ* of the edge-curve *λ* defined in Figure 1 can give us information about the non-trivial correspondence between the translation and the change of *Ω*. The slope function *σ* is bounded both from above and from below (0.31 = *σ_1_<σ<σ_2_* = 0.77, see Figure 2), which means there are *(p, q)* values (*q/p<σ_1_*) for which punishment acts advantageously for cooperation in the whole *T-S* plane, but at the same time, still within the altruistic punishment region, there exist *(p, q)* values (*σ_2_*<*q/p*<1) for which cooperation is clearly set back. As the slope changes along the line, as shown explicitly in Figure 2, for intermediate punishment values the translation can influence the measure of cooperation differently at distinct points of the *T*-*S* plane.

The slope provides, at any point, information only about the direction of the translation vector; however, its length is also relevant, in particular in the intermediate region referred to above. Indeed, in this region altruistic punishment can tip the balance and change the winning strategy depending on the location in the *T*-*S* plane.

**Figure 3:**
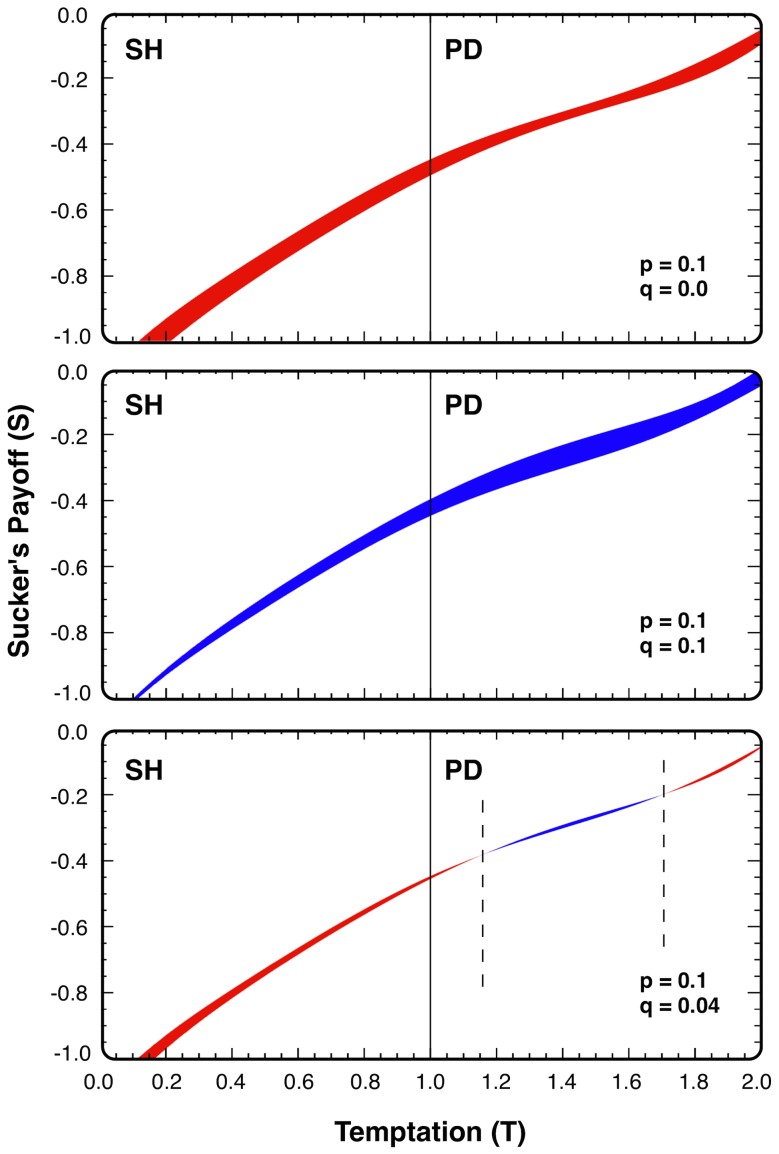
The effect of different *p*, *q* parameters on cooperation on the *T - S* plane. Upper panel (*q = 0*, *p = 0.1*): the introduction of punishment made cooperation dominant for an additional region (red shaded area). Middle panel (*q = 0.1*, *p = 0.1*): the ‘unsuitable’ use of punishment made a big domain of the *T - S* plane inaccessible for cooperation (blue shaded area). Lower panel (*q = 0.04*, *p = 0.1*): the effects of punishment are ambiguous: there are (*T, S*) values for which punishment enhances the overall cooperation (red shaded area); however on other areas it hinders it (blue shaded area located between vertical dashed lines).

Figure 3 depicts the change in the evolutionary outcome of cooperation for the three different scenarios identified above, showing that the additional costs of punishment can do more harm (blue areas in Figure 3) than good (red areas in Figure 3) to overall cooperation while in some cases the outcome is mixed. Although punishment contributes to reduce sizably the fitness of defectors, at the same time cooperators are burdened by the cost of inflicting this effect on their defective partners. This is especially true for hub players as they can be overburdened by the cost of punishing a huge number of defecting neighbors, which may result in a less cooperative outcome than without punishment. Eventually, the joint effect of two mechanisms that, each alone, help softening the social dilemma and promote cooperation, can interfere destructively and inhibit cooperation. This said, it is clear that for any fixed punishment value, there will exist a cost for which cooperation is enhanced. That is, if the cost of punishing the defecting individuals can be decreased, then the introduction of punishment may be a viable way to promote cooperation in network structured populations. Given the analysis above, however, not all combinations of *cost-punishment* will lead to a positive outcome. The principle can be summed up as: *Punish, but not at all costs*.

**Figure 4:**
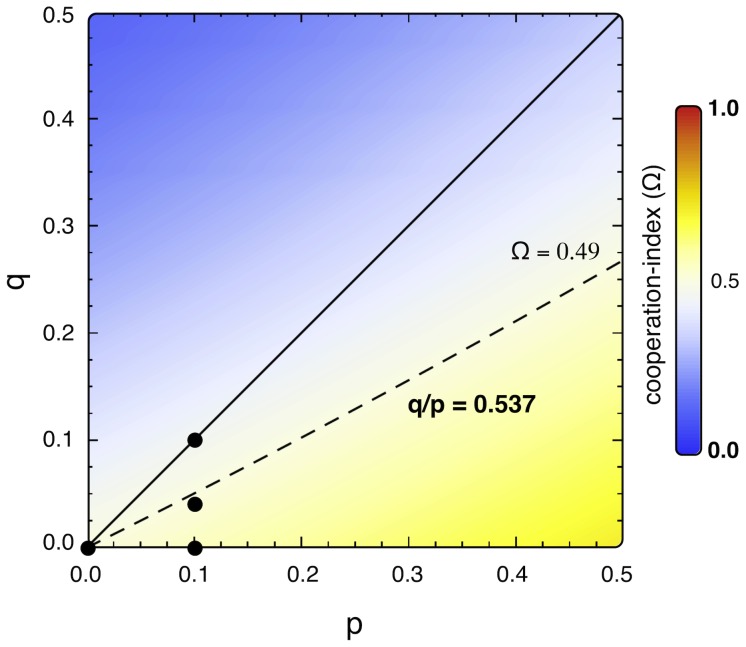
The overall impact on cooperation for different *p*, *q* parameters. The dashed line indicates cost-over-punishment ratio values for which overall cooperation remains unaffected by altruistic punishment (*Ω* = 0.49). The area below (above) the dashed line comprises the parameter region with enhanced (inhibited) cooperation. Black solid circles identify the parameter values used in the plots in Figures 1 and 3. Altruistic punishment (*p>q*) occurs below the solid line.

Figure 4 shows *Ω* for a wide range of *p* and *q* values. It can be seen that the regions with enhanced and diminished cooperation are clearly separated. The separation curve can be approximated very well by a straight line with slope *q/p* = 0.54. Qualitatively, this value can be considered as the average of the *σ* function displayed in Figure 2. Comparing the area of enhanced cooperation to that of diminished cooperation (in the parameter range *p>q*) of altruistic punishment), we observe that the introduction of altruistic punishment can decrease the overall cooperation level in a wide parameter region. It is worth noting that, in the limit of weak selection (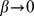), the network structure plays a minor role [62] in the overall evolutionary dynamics. As a result, the separation curve pictured as a solid line in Figure 4 becomes unambiguously straight (with a slope of 1), that is, for any *p>q*, *Ω* increases, in agreement with the analytical results obtained in ref. [64].

Naturally, the simple model proposed here does not provide an exhaustive analysis of the fate of altruistic punishment in structured populations. Important issues such as the role of anti-social punishment [65], [66] or the central issue of second-order free-riding [11] remain absent from our 2-strategy analysis. Concerning the latter, however, we have checked the evolutionary dynamics whenever individuals are allowed to choose between three strategies – cooperator (but not punisher), defector and punishing cooperator. As expected, the evolutionary dynamics becomes more complex in this case but the main results remain valid. The simulations are started from an initial state where all three strategies are equally represented in the population. Evolution always ends in a monomorphic state. Cooperators and punishers are neutral towards each other but even after the extinction of defectors, evolution is not governed by random drift; Hubs dictate the most likely evolutionary outcome of the population [67]. Cooperators and punishers can be considered as cooperative strategies in this more general setting. They both contribute to cooperation dominance in essentially 50% of the simulations. The identity of the winning strategy depends sensitively on the initial conditions, more specifically on the initial strategy of the hub-players. Regarding the average strategy distribution at the end of the evolutionary process, one can interpret it as the “superposition” of scenarios with and without punishment. In other words, the shift of the edge-curve *λ* in the case of 3 strategies is about half of what would be obtained in a scenario of defectors and punishers only, for the same parameter values. Overall, the three-strategy scenario exhibits qualitatively the same features as the two-strategy case analyzed in greater detail here.

To conclude, we study the impact of altruistic punishment in a population of individuals engaging in social dilemmas of cooperation where individuals can interact with each other alongside a structure described by a scale-free network. We find that depending on the *q/p* ratio between the cost to induce punishment and the actual extent of punishment, altruistic punishment can either enhance or inhibit cooperation. Mechanisms – such as structured populations and altruistic punishment – which separately promote cooperation, can have overall detrimental effects when applied together. This means that the introduction of punishment is not an easy question. The key to the success of punishment is to minimize the costs to be inflicted on those who engage in punishment. Indeed, only for low values of the *q/p* ratio will punishment effectively promote cooperation in networked populations. While from a well-mixed perspective punishment may seem a viable route towards cooperation [34], [35], [38], heterogeneous structured populations often narrow such pathway. In fact, and similar to what has been shown in the context of indirect reciprocity [68], the viability of punishment may be limited, such that it can be even easier to achieve cooperation in the absence of punishers whenever individuals interact in a realistic interaction setting.
